# Investigating the Influence of Mg Content Variations on Microstructures, Heat-Treatment, and Mechanical Properties of Al-Cu-Mg Alloys

**DOI:** 10.3390/ma16124384

**Published:** 2023-06-14

**Authors:** Abdul Wahid Shah, Seong-Ho Ha, Jabir Ali Siddique, Bong-Hwan Kim, Young-Ok Yoon, Hyun-Kyu Lim, Shae K. Kim

**Affiliations:** 1 Industrial Technology Department, University of Science and Technology, Daejeon 34113, Republic of Korea; abdulwahid.shah799@gmail.com (A.W.S.); jabirali94@kitech.re.kr (J.A.S.); bonghk75@kitech.re.kr (B.-H.K.); shae@kitech.re.kr (S.K.K.); 2 Industrial Materials Processing R&D Department, Korea Institute of Industrial Technology, Incheon 21999, Republic of Korea; veryoon@kitech.re.kr (Y.-O.Y.); hklim@kitech.re.kr (H.-K.L.)

**Keywords:** Al-Cu-Mg alloys, microstructure evolution, heat treatment, tensile properties

## Abstract

The objective of this study was to examine the impact of varying magnesium levels in the α-Al + S + T region of the Al-Cu-Mg ternary phase diagram on the solidification process, microstructure development, tensile properties, and precipitation hardening of Al-Cu-Mg-Ti alloys. The outcomes indicate that alloys with 3% and 5% Mg solidified with the formation of binary eutectic α-Al-Al_2_CuMg (S) phases, whereas in the alloy with 7% Mg, the solidification process ended with the formation of eutectic α-Al-Mg_32_(Al, Cu)_49_ (T) phases. Additionally, a significant number of T precipitates were noticed inside the granular α-Al grains in all alloys. In the as-cast condition, the 5% Mg-added alloy showed the best combination of yield strength (153 MPa) and elongation (2.5%). Upon T6 heat treatment, both tensile strength and elongation increased. The 7% Mg-added alloy had the best results, with a yield strength of 193 MPa and an elongation of 3.4%. DSC analysis revealed that the increased tensile strength observed after the aging treatment was associated with the formation of solute clusters and S″/S′ phases.

## 1. Introduction

Aluminum-silicon (Al-Si)-based casting alloys are widely utilized in the automobile industry for producing various powertrain components because of their excellent castability. However, they can exhibit relatively low strength, which further decreases at elevated temperatures. Because of this, these alloys are not suitable for high temperature applications in the aerospace, automotive, and other industries. Aluminum-copper-magnesium (Al-Cu-Mg) alloys containing high levels of magnesium, such as A240, 242, and 243, are often preferred for higher-temperature applications due to their excellent combination of strength, hardness, and wear resistance at both room and elevated temperatures [1,2,3,4,5]. High Mg-containing Al-Cu-Mg commercial alloys have limited elongation, typically less than 1%, which is significantly lower than the elongation exhibited by low Mg-containing Al-Cu-Mg alloys, such as A206, which typically have an elongation of 8–11% in the T7 temper [6,7,8,9,10,11,12,13,14,15]. As a result, these high Mg-containing alloys are typically only used in applications where thermal stability and/or wear resistance are the primary requirements [1,2,12,13,14,15,16,17,18,19]. These alloys, including A240, 242, and 243, are commonly used in aircraft engines and diesel engines due to their excellent thermal stability and wear resistance [1,2,3,4,5]. Despite their desirable thermal stability and wear resistance, these alloys cannot be utilized in applications that necessitate a favorable balance between strength and elongation due to their low elongation. Therefore, further investigation of the Al-Cu-Mg ternary system is necessary to develop novel alloys that exhibit high strength, ductility, and improved castability.

On the aluminum-rich corner of the Al-Cu-Mg ternary phase diagram, multiple phases are in equilibrium with the primary α-Al phase, including Al_2_Cu (θ), Al_2_CuMg (S), and Al-Mg_32_(Al, Cu)_49_ (T) [2]. The S phase has an orthorhombic crystal structure (Cmcm space group) with a narrow homogeneity range and a density of 3.55 g/cm^3^. Its micro-hardness decreases from 4.44 GPa at 20 °C to 2.22 GPa at 300 °C. By contrast, the T phase (~25% Cu, ~28% Mg) has a defective bcc crystal structure with a space group of Im3 and is isomorphic to the Al_2_Mg_3_Zn_3_ phase. It has a slightly higher density (4.14 g/cm^3^) and lower micro-hardness (4.14 GPa) than the S phase at both room and elevated temperatures [2]. Commercially available Al-Cu-Mg alloys mainly exist in the Al + S + θ section of the Al-Mg-Cu ternary phase diagram [1,2,16,17,18,19,20,21,22].

Commercial Al-Cu-Mg alloys, specifically those with less than 1% Mg content (such as A201 and A206), demonstrate exceptional strength and toughness through precipitation hardening. Nevertheless, these alloys possess certain drawbacks, including a relatively high susceptibility to hot tearing and low resistance to corrosion [1,2,7,8,9]. Conversely, alloys with higher Mg content (e.g., 240, 242, and 243) exhibit improved fluidity and hot tearing resistance but suffer from very low elongation, rendering them unsuitable for many applications. Consequently, the utilization of Al-Cu-Mg alloys is significantly limited compared to Al-Si-based casting alloys. Hence, there is a need to develop novel Al-Cu-Mg alloys that possess high strength, ductility, and improved castability. In this regard, the current focus lies in exploring compositions within the Al + S + T region of the ternary phase diagram. To the authors’ knowledge, no prior attention has been given to the development of Al-Cu-Mg alloys situated in the Al + S + T section of the ternary phase diagram. Therefore, the current study is the first to report on the microstructure evolution, heat treatment response, and mechanical properties of compositions located within the Al + S + T section of the phase diagram.

The aim of this research was to investigate how changes in the Mg composition in the Al + S + T region of the Al-Mg-Cu ternary system affect the solidification process, development of microstructure, mechanical characteristics, and precipitation hardening of Al-Cu-Mg-Ti alloys.

## 2. Materials and Methods

The chemical composition of the examined alloys employed in the investigation is presented in Table 1. The addition of Mg, Cu, and titanium (Ti) to the melts was accomplished using Mg + Al_2_Ca, Al-50% Cu, and Al-5% Ti-1% B master alloys (all compositions in mass%), respectively. Once the pure aluminum with 99.99% purity had melted, the alloying process was conducted at a temperature of approximately 770~800 °C. The gas bubbling filtration (GBF) process was utilized for 15 min to eliminate oxide inclusions and dissolved hydrogen gas using Ar gas. Following the melt treatment, the temperature of the melts was maintained at approximately 690 °C for 5 min before being poured into a steel mold that had been preheated to 200 °C. The chemical composition of developed alloys was examined using optical emission spectroscopy (OES, Bruker model Q2 ION, Bruker, Billerica, MA, USA).

The experimental alloys underwent a heat treatment process that began with a solution heat treatment at 477 °C for a duration of 10 h. The samples were then water-quenched and subjected to aging treatment at different temperatures (170 °C and 200 °C) for 20 h. To determine the change in hardness with the aging time, the Brinell hardness machine (Buehler, Uzwil, Switzerland) was employed. Five hardness values were obtained for each specimen at each condition, and the average of these five values is presented.

The microstructure was observed using optical microscopy (OM, Nikon, Tokyo, Japan) and field emission-scanning electron microscopy (FE-SEM, FEI model Quanta 200 F, Hillsboro, OR, USA) with energy dispersive spectroscopy (EDS, EDAX, Pleasanton, CA, USA). Before optical microscopy (OM) was utilized to observe the microstructure, the samples underwent preparation involving grinding and micro-polishing, followed by etching in Keller’s reagent. The FE-SEM analysis was carried out under specific conditions, including an accelerating voltage of 20 KV and a working distance of 10.0 mm. The volume fraction of eutectic phases within each alloy was determined using the ImageJ software (version 1.51). Phase analysis was conducted using X-ray diffraction (XRD D8 Discover) with a scanning step size of 0.02 degrees and a speed of 0.5 s per step. The XRD patterns were analyzed using the EVA software program (version 11.0). The samples were prepared in accordance with ASTM standard B557 for tensile testing, and a universal tensile testing machine (DTU-900MHN, Daekyung Tech, Gumisi, Republic of Korea) was used to perform the tests. Throughout all of the tests, a strain rate of 1.5 mm/min was utilized, and the gauge length of the extensometer was 30 mm. The differential scanning calorimetry (DSC, TA Q1000 instrument, TA instruments, Milford, MA, USA) experiments involved heating each alloy’s sample at a rate of 10 °C per minute under an argon atmosphere between 100 °C and 700 °C. Theoretical calculations were carried out using *Fact-Sage 7.1* software.

## 3. Results and Discussion

**Figure 1:**
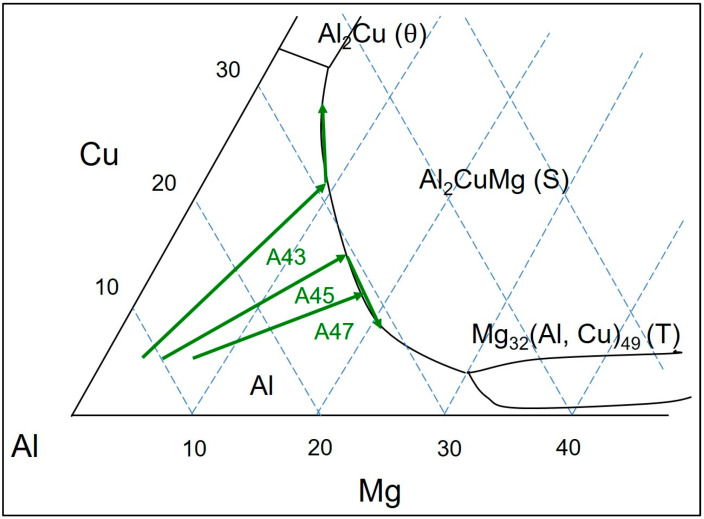
The aluminum-rich corner of the ternary liquid projection phase diagram of the Al-Cu-Mg system.

**Figure 2:**
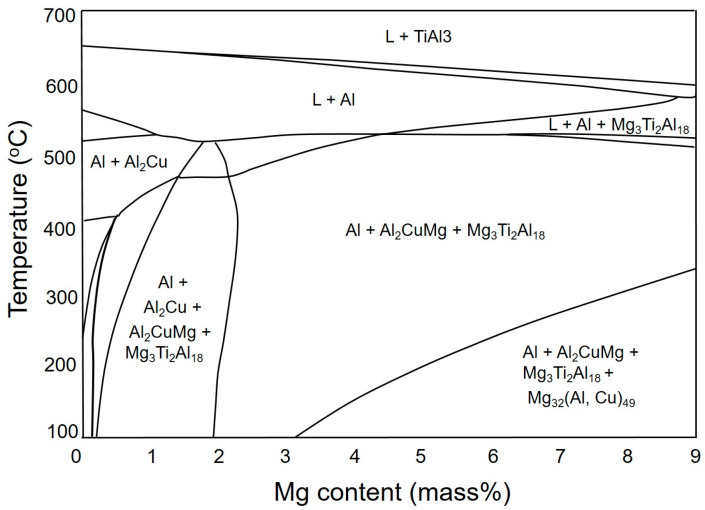
The vertical section of the phase diagram for the Al-4.5 wt% Cu-0.2 wt% Ti-0~9 wt% Mg alloy system.

In the Al-Mg-Cu ternary phase diagram, the Al_2_CuMg intermetallic compound acts similarly to Mg_2_Si in the Al-Mg-Si ternary system, dividing the diagram into two regions in the Al-rich corner, which are (1) α-Al + θ + S and (2) α-Al + T + S (as indicated in Figure 1). In the Al-Cu-Mg ternary system with a Cu/Mg ratio of 2.40, a binary eutectic reaction (L ≥ α-Al + S) takes place, leading to several non-variant reactions in the Al-rich corner of the alloys [2]. FactSage calculations, based on the Scheil model, indicate that the Al + S eutectic phases in the Al-4.5Cu-3.0Mg alloy form at temperatures between 529 °C and 517 °C, after which Al + S + θ eutectic phases are formed at 517 °C, representing the solidus temperature of the alloy. By contrast, in the Al-4.5Cu-5.0Mg and Al-4.5Cu-7.0Mg alloys, it is predicted that the formation of Al + S eutectic phases will occur until 468 °C, after which Al + T eutectic phases are formed, followed by the formation of Al-T-Al_3_Mg_2_ eutectic phases at 450 °C. Figure 2 shows that the final microstructure of the AlCu4.5Ti0.2Mg3 (A43) alloy is predicted to consist of α-Al, S, and Al18Ti2Mg3 phases. Moreover, when the Mg content increases to 5% and 7% in AlCu4.5Ti0.2Mg5 (A45) and AlCu4.5Ti0.2Mg7 (A47) alloys (respectively), theoretical calculations suggest that T phases (in addition to S- and Ti-based phases) will precipitate out when the temperature decreases to a certain value, depending on the Mg content.

**Figure 3:**
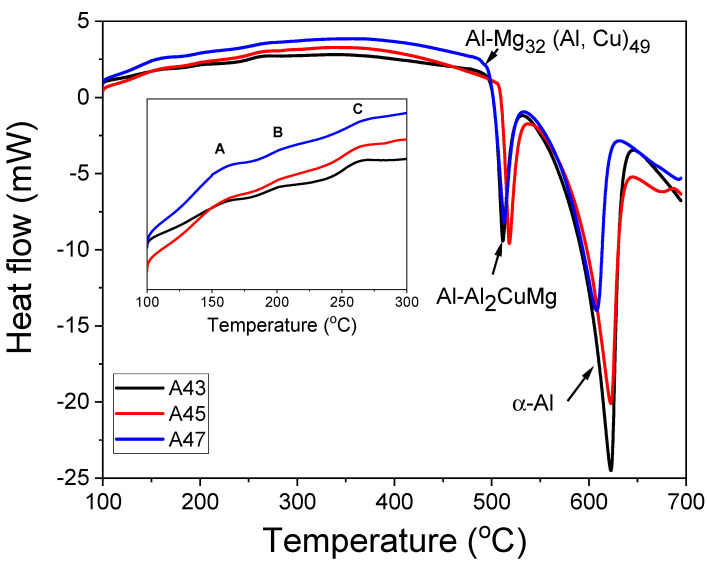
Differential scanning calorimetry (DSC) thermograms of the investigated alloys, which were obtained by heating at a rate of 10 °C per minute.

In Figure 3, DSC thermographs of these alloys in the as-cast temper are presented. The thermographs showed two distinct peaks, one indicating the development of primary α-Al phases and the other representing the eutectic α-Al-S phases. Additionally, the thermograph of the A47 alloy showed a peak associated to eutectic α-Al-T phases. The results indicate that the solidification process concluded with a binary eutectic reaction (L ≥ α-Al + S) in the A43 and A45 alloys, while the L + S ≥ α-Al + T reaction completed the solidification in the A47 alloy at a lower solidus temperature than the former alloys [2].

**Figure 4:**
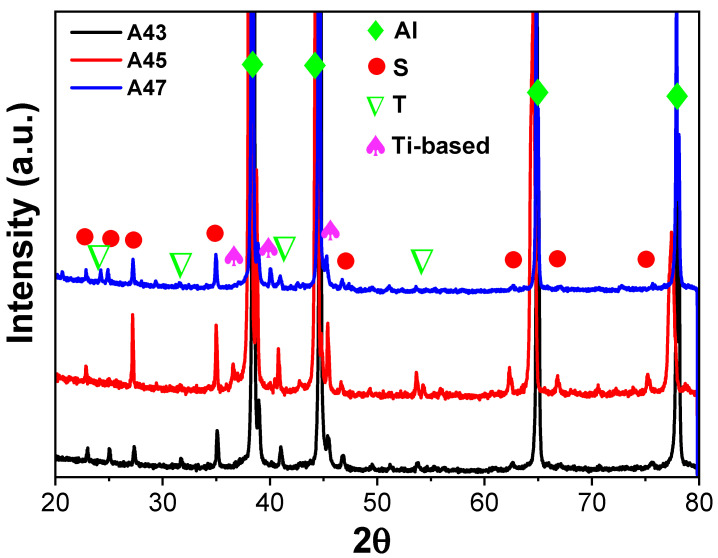
XRD patterns of the as-cast investigated alloys.

Furthermore, all alloys displayed exothermic peaks between 100 and 300 °C, where peak A was likely to indicate the precipitation of T phases, which is consistent with the phase diagram shown in Figure 2. Surprisingly, all alloys exhibited formation temperatures that were almost similar, in contrast to theoretical calculations. The next exothermic peak B could be connected to the creation of solute clusters. Previous studies suggested that the peak C observed between 250 and 280 °C could potentially be linked to the precipitation of S″ or S′ phases [23,24,25,26]. The X-ray diffraction (XRD) results of the examined alloys support the conclusions derived from the DSC analysis. The XRD patterns exhibited peaks corresponding to primary aluminum, Al_2_CuMg (S), Mg_32_ (Al, Cu)_49_ (T), and Ti-based phases in all the investigated alloys, as illustrated in Figure 4. This indicates that, similar to the DSC analysis, the XRD analysis has also confirmed the presence of T precipitates within the A43 alloy, as well as in the other alloys.

Table 2 compares the solidification ranges of the investigated alloys, as obtained from both theoretical calculations and experimental results (Figure 3). As the Mg content increased, there was a notable reduction in the liquidus temperature. According to equilibrium-based theoretical calculations, the A43 alloy was predicted to have a solidification range of 113.8 °C, which increased to 171.4 °C and 157.9 °C in the A45 and A47 alloys, respectively. However, the DSC results showed that the A43 alloy had a solidification range of 134 °C, which decreased significantly in the A45 alloy to 113 °C, before slightly increasing again to 119.5 °C in the A47 alloy. The reason why the A47 alloy has a wider range of freezing compared to the A45 alloy may be due to the creation of eutectic α-Al-Mg32(Al,Cu)49 phases.

**Figure 5:**
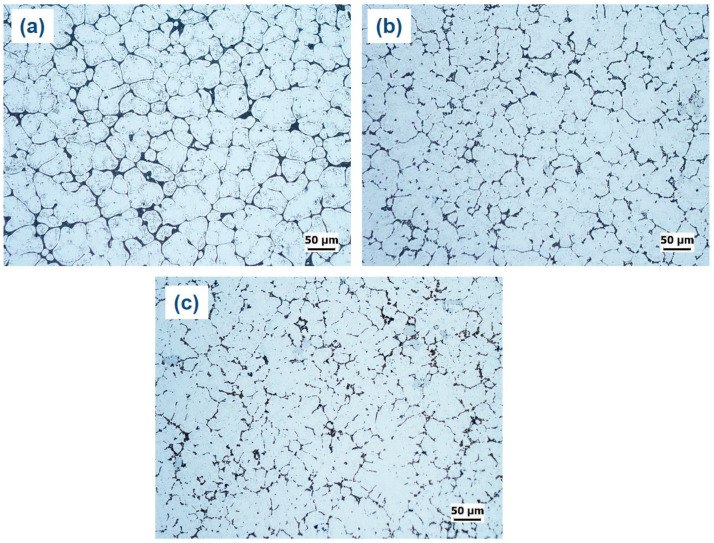
Optical micrographs of as-cast investigated alloys: (**a**) A43, (**b**) A45, and (**c**) A47.

**Figure 6:**
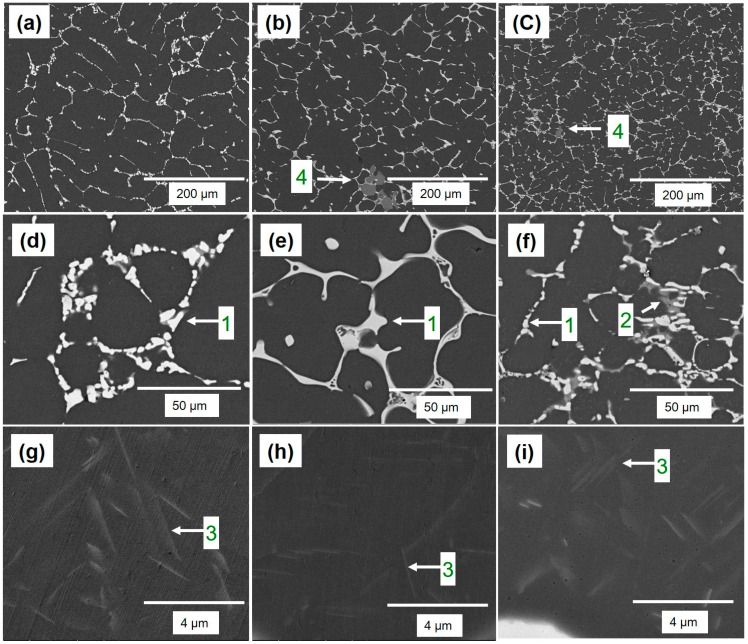
FESEM-micrographs of the as-cast A43, A45, and A47 alloys, with (**a**–**g**), (**b**–**h**), and (**c**–**i**) corresponding to each alloy, respectively. In each micrograph, 1, 2, 3, and 4 are used to indicate the eutectic Al-S, eutectic Al-T, T- precipitates, and Ti-based intermetallic, respectively.

**Figure 7:**
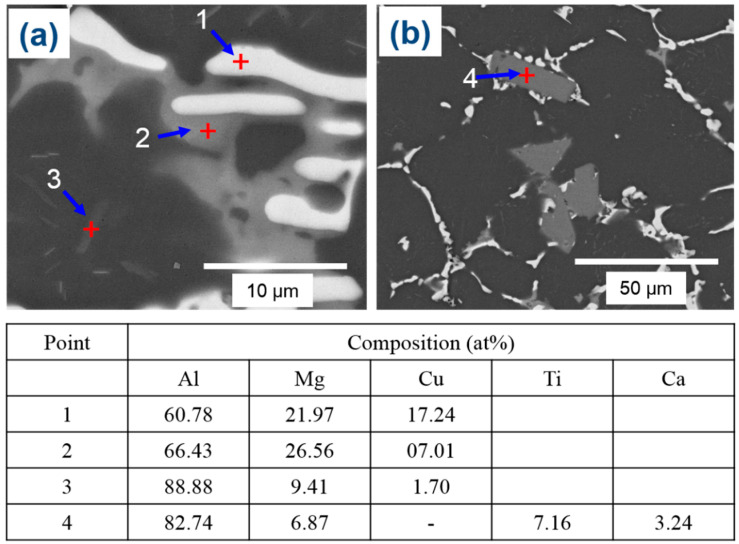
FESEM-micrographs (**a**,**b**) of the A47 alloy in its as-cast condition, while the corresponding compositions of the points indicated in the micrographs are given in the table below.

Figure 5 illustrates that the α-Al matrix in all the analyzed alloys had a globular grain structure, with eutectic α-Al-S phases located at the boundaries of the grains and T precipitates present within the globular grains. Additionally, the A45 and A47 alloys showed the presence of Mg_3_Ti_2_Al_18_ intermetallic compounds (Figure 6). The FESEM-micrographs of the alloys are presented in Figure 6, with the eutectic α-Al-S, eutectic α-Al-T, T precipitates, and Mg_3_Ti_2_Al_18_ intermetallic compound denoted as 1, 2, 3, and 4, respectively. Figure 7 displays the EDS analysis results that were used to determine the exact composition of the second phases. During the solidification process, the T phases were seen to precipitate when the temperature dropped below the solidus temperature, as revealed by the DSC results in Figure 3.

**Figure 8:**
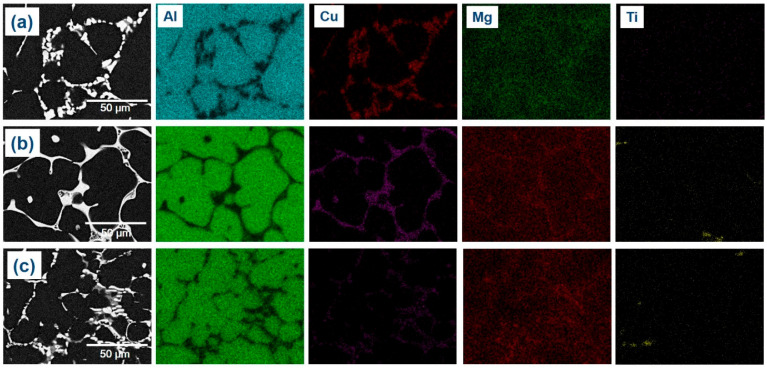
FESEM-micrographs and the corresponding mapping analysis results of the A43 (**a**), A45 (**b**), and A47 (**c**) alloys.

The experimental findings presented in Figure 7 are consistent with the theoretical calculations shown in Figure 2. However, there were some discrepancies between the experimental and theoretical results. For example, T phases were detected in the A43 alloy, despite not being predicted by the theoretical calculations. Additionally, Mg_3_Ti_2_Al_18_ intermetallic compounds were also observed in all alloys, which was confirmed by EDS mapping analysis (Figure 8). This finding aligns well with the XRD results depicted in Figure 4. EDS analysis (Figure 7) also revealed a considerable quantity of Ca in the Mg_3_Ti_2_Al_18_ intermetallic compounds. The A47 alloy also exhibited eutectic α-Al-T phases in addition to the eutectic α-Al-S phases, which is consistent with the peak related to this reaction (L + S ≥ α-Al + T) observed in the DSC thermograph (Figure 3). The eutectic morphology differed among the alloys, with the A43 and A47 alloys displaying rounded eutectic α-Al-S phases, while the A45 alloy had interconnected eutectic phases.

**Figure 9:**
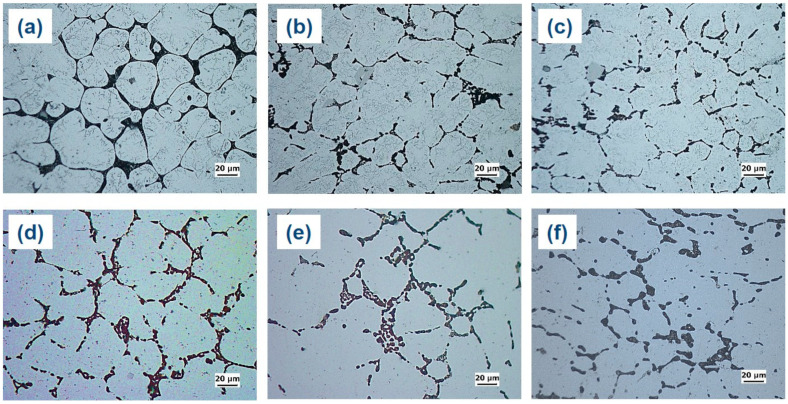
Optical micrographs of the investigated alloys in different temper conditions. Specifically, (**a**–**c**) depict the microstructures of the A43, A45, and A47 alloys, respectively, in their as-cast condition, while (**d**–**f**) reveal the microstructures of the A43, A45, and A47 alloys, respectively, in their as-quenched condition.

The microstructures of the as-cast and as-quenched alloys are compared in Figure 9. It can be seen that the eutectic α-Al-S phases are still present in the as-quenched alloys, indicating that the solution heat treatment did not significantly modify these phases. Similarly, the Mg_3_Ti_2_Al_18_ intermetallic compounds remained almost unchanged after the solution treatment. Nevertheless, the solution heat treatment led to a substantial dissolution of T precipitates within the globular grains of primary Al, which suggests that the T precipitates have a greater solubility in the Al matrix at higher temperatures.

**Figure 10:**
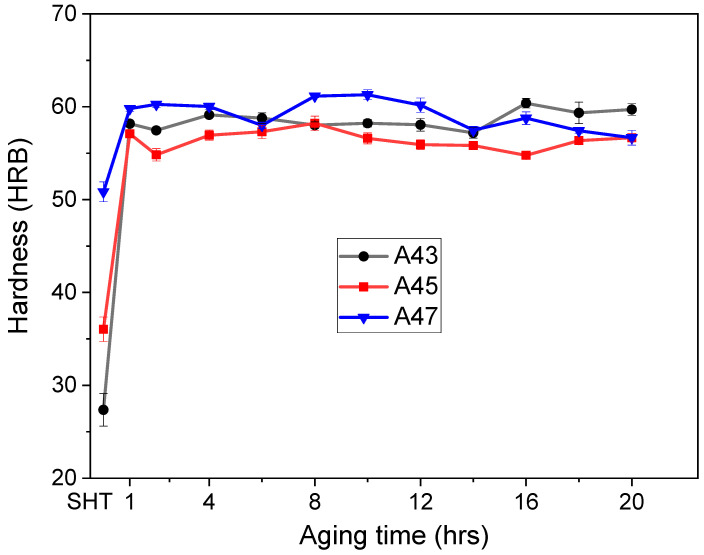
The evolution of hardness values for the examined alloys as a function of aging time at 170 °C.

**Figure 11:**
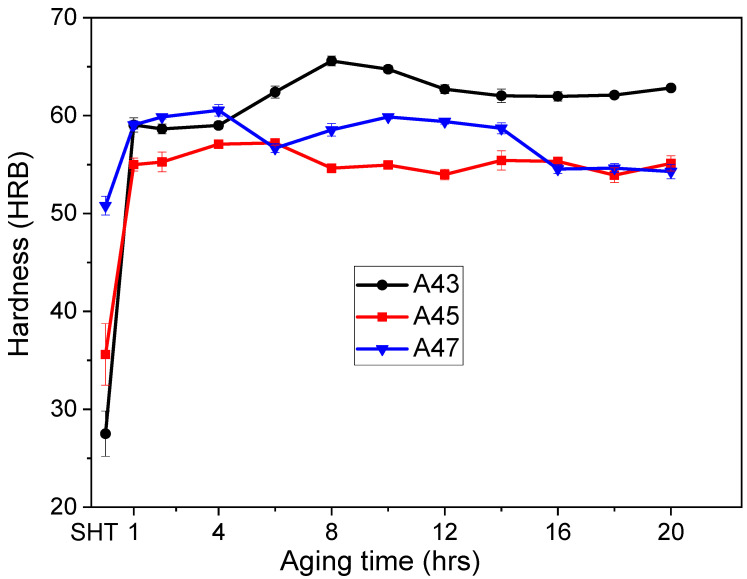
The evolution of hardness values for the examined alloys as a function of aging time at 200 °C.

Figure 10 and Figure 11 display how the hardness values of the alloys changed over time at various aging temperatures. The as-quenched hardness values were different for the three alloys, with A47 exhibiting the highest initial hardness value of 51 HBR, followed by A45 with a value of 36 HBR, and A43 with the lowest value of 27 HBR (Figure 9). The increment in hardness values after aging was not the same for all alloys, and it was observed to be dependent on the Mg content. The rate of hardness increase was slower for the A47 alloy than for the A43 and A45 alloys, which showed a rapid increase in hardness after 1 h of aging, followed by a slight decrease before increasing gradually up to 10–12 h. Following that time, the hardness values remained relatively constant for the remainder of the aging duration. Previous studies [23,24,25,26] on Al-Mg-Cu alloys with higher Mg contents have identified two distinct peaks in the evolution of hardness during aging. The first peak in hardness is associated with the creation of atomic clusters or GPB zones, which contribute to as much as 60% of the overall hardening that takes place during aging. The second stage of precipitation hardening, which happens later in the aging process, corresponds to the precipitation of S″ and S′ phases. Similar findings were observed in this study for the current alloys, where more than 60% of total hardness was achieved after 1 h of aging. Moreover, when aged at a higher temperature of 200 °C, a similar trend was observed, but A43 alloy showed a considerable increase in hardness, and a peak hardness of 65 HRB was obtained after 8 h of aging (Figure 11). In conclusion, the hardness values of the alloys significantly increased after aging, but the rate and magnitude of increase varied with the magnesium content and aging temperature.

**Figure 12:**
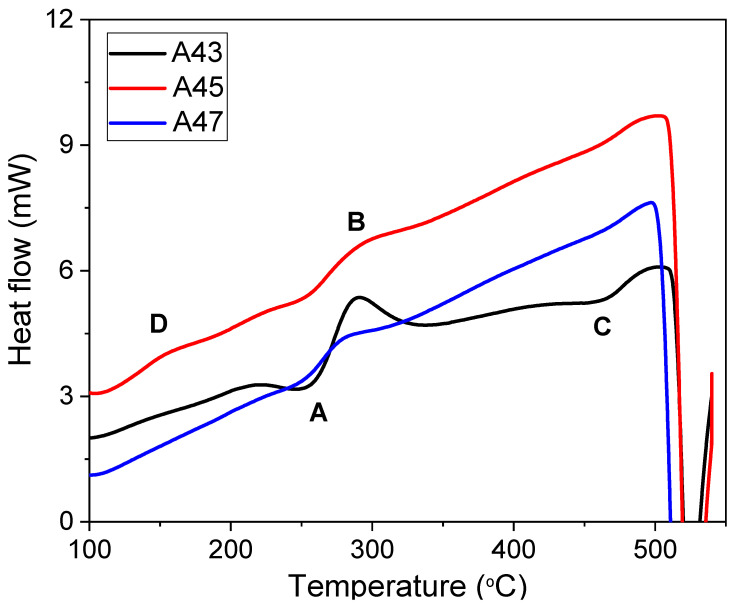
Differential scanning calorimetry (DSC) curves of the investigated alloys when heated from 25 °C to 550 °C under argon atmosphere at a rate of 10 °C per minute.

In their as-quenched state, Al-Cu-Mg alloys are made up of a supersaturated solid solution (SSSS), which is a high-energy state [23,24,25,26]. During precipitation hardening, this state transforms into solute clusters as the first stage. The growth of these solute clusters regions causes the creation of the S″ metastable phase, which eventually transforms to another S′ (metastable phase) over time. The stable S phase is formed through the growth of the metastable S′ phase. Figure 12 displays the DSC thermograms of the investigated alloys in their as-quenched tempers, revealing two endothermic peaks (A and C) and one exothermic peak (B) common to all alloys. Previous studies [23,24,25,26] have associated endothermic peak A with the dissolution of solute clusters, while exothermic peak B indicates the precipitation of S″ or S′ phases [25]. The DSC curves of A45 and A47 alloys showed a significant decrease in the area of exothermic peak B compared to that of A43, indicating a reduction in precipitation hardening in A45 compared to A43. Peak C represents the dissolution of S″ or S′ phases, and an additional exothermic peak (D) was observed in the A45 alloy, which may be attributed to the formation of T precipitates, as discussed previously. These results demonstrate that the kinetics of precipitate formation vary significantly with changes in the Mg/Cu ratio, leading to substantial differences in the hardness changes with time observed for these alloys, as illustrated in Figure 10 and Figure 11.

**Figure 13:**
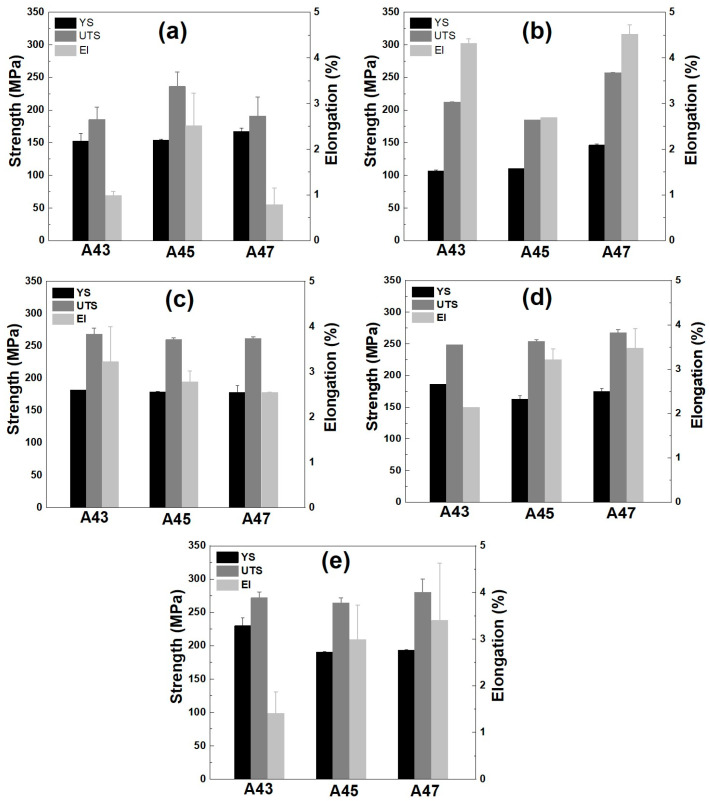
Tensile properties of the investigated alloys in different tempers, including (**a**) as-cast, (**b**) as-quenched, (**c**) T6 temper-aged at 170 °C for 1 h, (**d**) T6 temper-aged at 200 °C for 1 h, and (**e**) T6 temper-aged at 200 °C for 8 h.

Figure 13 and Table 3 present the tensile properties of these examined alloys under different conditions. In the as-cast state, the A43 and A45 alloys showed similar yield strength values of around 150 MPa (Figure 13a), but the A45 alloy showed a higher elongation of 2.5%, indicating a better combination of strength and ductility compared to commercial alloys [1,2,3]. The enhanced elongation of the A45 alloy might be attributed to the variation in the morphology of the eutectic phases present in this alloy compared to other alloys (Figure 6). Additionally, the higher Mg content in the A47 alloy increased the yield strength to 167 MPa but reduced the elongation to 0.76%. The increase in yield strength with increasing Mg content is likely due to the increasing amount of eutectic phases in the A47 alloy. The volume fraction of eutectic phases was approximately 6.7% in the A43 alloy. This fraction increased to 9.5% in the A45 alloy and further to 15.1% in the A47 alloy. The overall tensile strength of the as-cast alloys is expected to have been contributed to by grain boundary strengthening from the granular primary Al matrix and strengthening induced by eutectic phases and T precipitates (via thermal mismatch, load transfer, and Orowan looping). Following solution heat treatment, the yield strength of the alloys decreased, while the ultimate tensile strength and elongation increased (Figure 13b). Enhanced elongation in the as-quenched state of these alloys may arise from the T phases dissolving within the aluminum matrix, a uniform dispersion of solute atoms, and the rounding of eutectic phases. Additionally, the A47 alloy showed a higher yield strength of 146 MPa, which aligns with the hardness findings presented in Figure 10 and Figure 11. The increased yield strength observed in the A47 alloy can be attributed to its larger quantity of remnant phases in the as-quenched state compared to the other alloys. This higher concentration of remnant phases can be directly linked to the fact that the A47 alloy had a greater amount of eutectic phases in the as-cast condition. Aging treatments for 1 h at 170 °C (Figure 13c) and 200 °C (Figure 13d) resulted in a significant improvement in tensile strength for all alloys along with an increase in elongation. A longer exposure of 8 h at 200 °C further improved yield strength in all alloys with no significant impact on elongation (Figure 13e). The improvement in the strength of the aged samples compared to the as-quenched tempers can be attributed to precipitation strengthening induced by the formation of solute clusters and the creation of S″ or S′ phases. The A47 alloy exhibited the best results, with a yield strength of 193 MPa and an elongation of 3.4%, surpassing the yield strength–ductility combinations achieved in commercially available high Mg-added Al-Cu-Mg alloys (e.g., 240, A242, and 243) used for aircraft engine parts [1,2,3].

## 4. Conclusions

The aim of this research was to investigate how changes in the Mg composition in the α-Al + S + T region of the Al-Mg-Cu ternary phase diagram affect the solidification process, development of microstructure, precipitation hardening, and mechanical characteristics of Al-Cu-Mg-Ti alloys. The outcomes indicated that alloys with 3% and 5% Mg solidified with the creation of binary eutectic α-Al-Al_2_CuMg (S) phases, whereas in the alloy with 7% Mg, the solidification process ended with the creation of eutectic α-Al-Mg_32_(Al, Cu)_49_ (T) phases. Additionally, a significant number of T precipitates were noticed inside the granular α-Al grains in all alloys. In the as-cast condition, the 5% Mg-added alloy showed the best combination of yield strength (153 MPa) and elongation (2.5%). Upon T6 heat treatment, both tensile strength and elongation increased. The 7% Mg-added alloy had the best results, with a yield strength of 193 MPa and an elongation of 3.4%. These mechanical properties have not been previously observed in commercially available Al-Cu-Mg alloys with high Mg content. DSC analysis revealed that that the increased tensile strength observed after the aging treatment was associated with the formation of solute clusters and S″/S′ phases.

## Figures and Tables

**Table 1:** Nominal and analyzed compositions of the alloys that were investigated.

Alloy	Nominal Composition (Mass %)	Analyzed Compositions (Mass %)					
		Cu	Mg	Ti	Fe	Si	Al
A43	AlCu4.5Ti0.2Mg3	4.30	3.13	0.263	0.0964	0.022	bal.
A45	AlCu4.5Ti0.2Mg5	4.11	5.23	0.287	0.0942	0.010	bal.
A47	AlCu4.5Ti0.2Mg7	4.09	7.44	0.267	0.0952	0.00087	bal.

**Table 2:** Liquidus and solidus temperatures, as well as the corresponding solidification range, which were determined through theoretical calculations and differential scanning calorimetry (DSC) analysis.

Alloy	Theoretical Calculations			DSC Analysis		
	T_L_	T_S_	ΔT	T_L_	T_S_	ΔT
A43	631.54	517	113.8	635.03	500.32	134.71
A45	621.92	450.46	171.4	623.03	509.73	113.3
A47	608.38	450.46	157.9	607.7	488.04	119.6

**Table 3:** Tensile properties of the alloys that were examined in both their as-cast and heat-treated conditions.

Temper	Alloy	Yield Strength (MPa)	Ultimate Tensile Strength (MPa)	Elongation (%)
As-cast	A43	152.59 ± 11.9	185.55 ± 18.9	0.985 ± 0.09
	A45	153.79 ± 1.8	236.21 ± 21.8	2.515 ± 0.71
	A47	167.27 ± 5.03	191.0 ± 28.9	0.79 ± 0.36
As-quenched	A43	107 ± 1.4	212.51 ± 0.2	4.32 ± 0.1
	A45	110.7 ± 0.0	184.9 ± 0.0	3.70 ± 0.0
	A47	146.36 ± 1.9	257.4 ± 0.5	4.52 ± 0.2
T6 (170 °C × 1 h)	A43	181.92 ± 0.00	268.36 ± 8.96	3.22 ± 0.77
	A45	179.13 ± 0.03	259.64 ± 2.59	2.78 ± 0.24
	A47	178.23 ± 10.4	261.57 ± 2.10	2.54 ± 0.01
T6 (200 °C × 1 h)	A43	186.29 ± 0.00	248.69 ± 0.00	2.14 ± 0.00
	A45	162.82 ± 4.82	253.73 ± 3.17	3.22 ± 0.24
	A47	174.90 ± 4.70	267.61 ± 4.84	3.47 ± 0.44
T6 (200 °C × 8 h)	A43	230.15 ± 11.6	271.91 ± 8.97	1.41 ± 0.46
	A45	190.68 ± 0.34	264.3 ± 7.24	2.99 ± 0.74
	A47	193.23 ± 0.70	280.13 ± 19.74	3.4 ± 1.23

## Data Availability

Available upon request from the corresponding author.
